# The effect of polyaniline on TiO_2_ nanoparticles as anode materials for lithium ion batteries

**DOI:** 10.1186/s40064-016-1908-z

**Published:** 2016-05-17

**Authors:** Haitao Zheng, Ntombizodwa M. Ncube, Kumar Raju, Nonhlanhla Mphahlele, Mkhulu Mathe

**Affiliations:** Material Science and Manufacturing, Council for Scientific and Industrial Research (CSIR), PO Box 395, Pretoria, 0001 South Africa

**Keywords:** TiO_2_, Polyaniline, Composite, Anode, Lithium ion battery

## Abstract

Polyaniline (PANI) additives have been shown to have a significant effect on titanium dioxide (TiO_2_) nanoparticles as lithium ion battery anode materials. TiO_2_/PANI composites were prepared using a solid coating method with different ratios of PANI and then characterized using XRD and SEM. These composites have shown increased reversible capacity compared with pure TiO_2_. At the current rate of 20 and 200 mAg^−1^, maximum capacities were also found on 15 % PANI incorporated TiO_2_ composite with 281 mAh g^−1^ and 168.2 mAh g^−1^, respectively, and 230 and 99.6 mAh g^−1^ were obtained in the case of pure TiO_2_. Among all the composite materials, 10 % PANI incorporated TiO_2_ composite exhibited the highest reversible capacity with cycle stability after 100 cycles at the current rate of 200 mAg^−1^, suggestive that the optimal ratio is 10 % PANI of TiO_2_/polyaniline. The cycle stability showed swift fade when the ratio of PANI in the composites exceeded 10 % though the highest initial capacity was achieved on 15 % PANI in the composites. These results suggest that PANI has effectively enhanced the reversible capacity of commercial TiO_2_, and may be a promising polymer matrix materials for lithium ion batteries.

## Background

Titanium based oxides have attracted tremendous attention from researchers as the potential next generation anode materials in lithium ion batteries. They are currently being investigated as potential graphite substitutes. The theoretical capacity of titanium dioxide (TiO_2_) is 330 mAhg^−1^ which is slightly lower than that of graphite at 372 mAhg^−1^. TiO_2_ also offers better properties over graphite owing to its high lithium insertion/de-insertion potential, higher reversible capacity and lower volume expansion during lithium ion insertion/de-insertion. This leads to enhanced structural stability and a longer cycle stability (Wang et al. 2007; Nuspl et al. 1997; Su et al. 2012). However, the practical attainable capacity of TiO_2_ is only half the theoretical value due to the blocking of further li-ion insertion of TiO_2_ resulting from the strong repulsive force between Li ions. This reportedly limits the application and development of TiO_2_ as anode materials for LIBs (Kavan et al. 1995; Kavan et al. 2000; Tang et al. 2009). Furthermore, its low conductivity is hampered for application of LIBs. Fortunately, it has been predicted by theoretical simulations with experimental results that the capacity and cycling stability of the TiO_2_ electrode can be improved dramatically when the nanoscale of TiO_2_ was explored (Sudant et al. 2005; Jiang et al. 2007; Fattakhova Rohlfing et al. 2007; Yang and Zeng 2004). The TiO_2_ performance of anode materials can be improved by combining TiO_2_ with other materials such as carbon (Yang et al. 2012; Wang et al. 2013).

Conducting polymers have drawn considerable attention due to their good environmental stability and electronic properties as well as their optical performance. The polymers have been studied as active matrices to improve the capacity, cycling stability and rate of performance of electrodes for LIBs because the polymer can provide a conducting backbone for the active materials amongst their many properties (Chen et al. 2012; Chew et al. 2007; Dong et al. 2013; Huang and Goodenough 2008; Jeong et al. 2013). Furthermore, with regard to electrochemical activity towards Li, the relatively inert matrix of polymeric composites would accommodate the mechanical stresses/strains resulting from the active phase which would maintain the structural integrity of the composite during lithium intercalation/de-intercalation. Polyaniline (PANI) is one of the more important conducting polymers because of its relatively facile processability, electrical conductivity and environmental stability. PANI has been studied extensively for energy storage systems because of its good redox reversibility and high stability (Novák et al. 1997; Karthikeyan et al. 2013; Liang et al. 2011). Mesoporous PANI/TiO_2_ microspheres were reported as anode materials with a significantly improved capacity (Lai et al. 2011; Lai et al. 2010). The material was calcined at 500 °C for 3 h in air. However, PANI begins to decompose around 300 °C and completely decomposes at 500 °C in air (Zeng and Ko 1998). We have repeated the same methods to prepare PANI/TiO_2_ composites without air treatment. In this work, we have prepared PANI/TiO_2_ composite materials using a mechanical method with a different ratio of PANI to TiO_2_ as anodes for lithium- ion batteries (Yang et al. 2008).

## Experimental

### Materials

Aniline, Ammonium peroxydisulfate and 37 % hydrochloric acid were purchased from Sigma-Aldrich. TiO_2_ and carbon black PRINTEX XE 2-B were procured from DUGASSA and used as received.

### Synthesis of polyaniline (PANI)

Polyaniline was synthesized by chemical polymerization. Aniline monomer was dissolved in the 0.02 M HCl aqueous solution and stirred magnetically at 0–5 °C for 0.5 h. An aqueous solution of (NH_4_)_2_S_2_O_8_ that acts as an oxidant was added to the above solution. The mixture was then left to react over night at 0–5 °C. The precipitate was washed with deionized water followed by methanol, and then finally dried overnight at 70 °C in a vacuum.

### Preparation of TiO_2_/polyaniline composite

TiO_2_/polyaniline composite was formed by the mechanical mixing method. The ratio of PANI/TO (w/w) was 0, 5, 10, 15 and 20 %, named as TO, TO5PA, TO10PA, TO15PA and TO20PA.

### Characterization

Electrochemical measurements were carried out between 1.0 and 3.0 V vs Li^+^/Li^0^ with CR2032 coin cells. The synthesized composites were mixed with Carbon black (PRINTEX XE 2-B) and PVDF (75:13:12 wt %) to fabricate the anode. In the coin-cell tests, metallic lithium foil was used as the counter and reference electrodes; the electrolyte was 1 M LiPF_6_ in 1:1 (v/v) solvent mixture of ethylene carbonate and diethyl carbonate (EC/DEC).

## Results and discussion

### SEM morphology

**Fig. 1:**
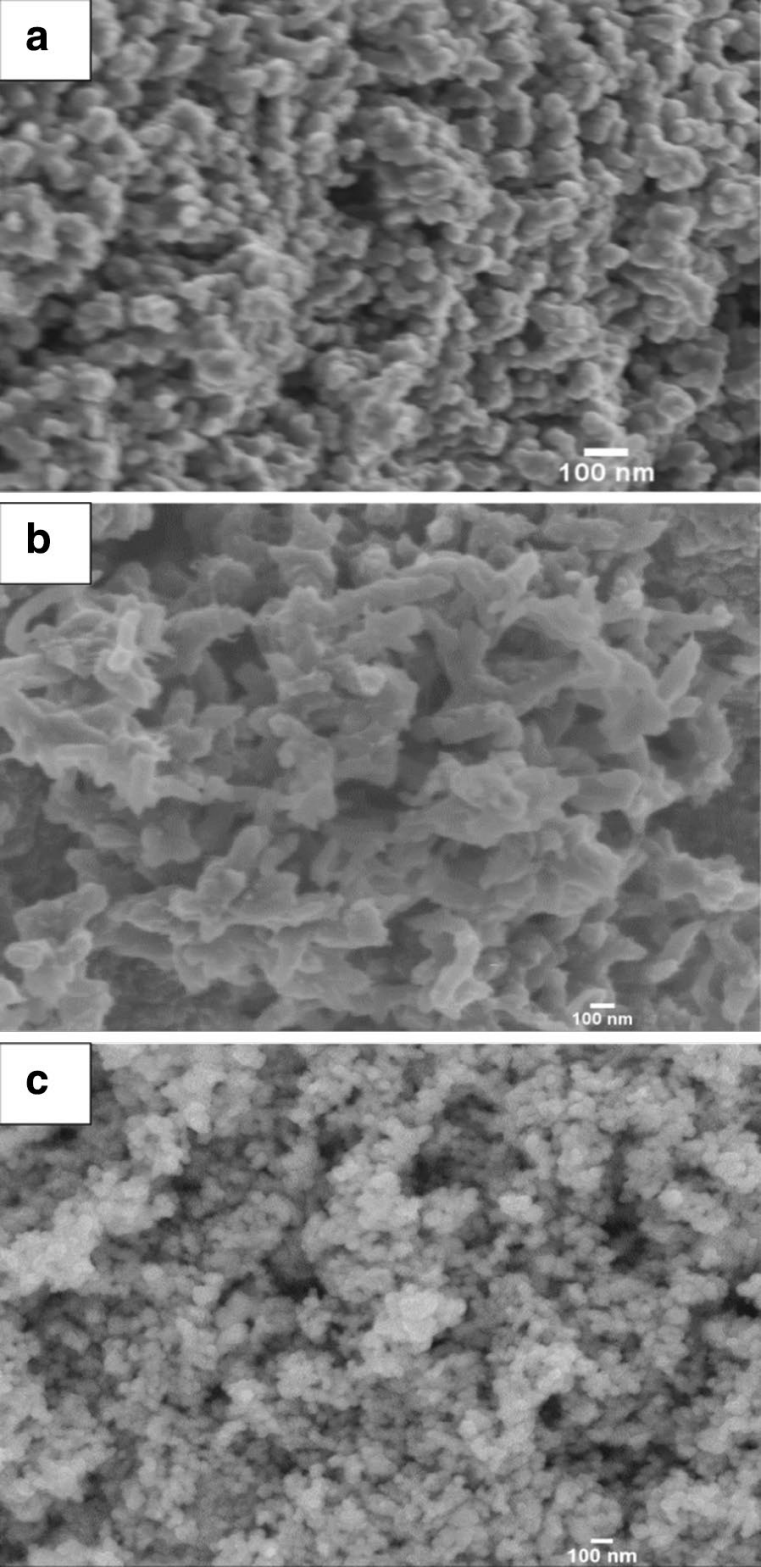
SEM images of TiO_2_ (**a**), PANI (**b**) and TO15PA (**c**)

Figure 1 shows the SEM micrographs of TiO_2_ (TO) and PANI. It can be seen from Fig. 1a that TO was made up of very uniform nanoparticles with the size distribution of 40 nm. Figure 1b shows that PANI was made up of nanorod with 45 nm diameter and 200-300 nm length. After grinding with PANI, no PANI nanorods were observed in Fig. 1c, which was probably because PANI nanorods were crashed and covered with TO nanoparticles.

### X-ray diffraction

**Fig. 2:**
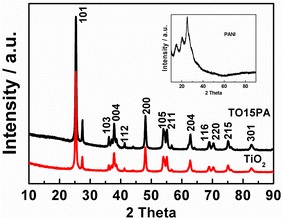
XRD patterns of TiO_2_, PANI (*inset*) and TO15PA composites

Figure 2 shows the XRD diffractograms of TO, PANI (inset) and the TO15PA composites. Typical XRD diffraction patterns of PANI revealed representative polyaniline peaks at 2θ = 14.9°, 20.4° and 25.0°. All the observed diffraction peaks matched the anatase TO phase crystal structure (Yang et al. 2008). However, there is no typical XRD patterns of PANI in TO15PA reported in literature; only the diffraction peaks of TO that appeared in the TO15PA sample compared with other patterns of TO. This might be related to the lower PANI amount in TO15PA that leads to diffraction peaks of PANI merging with the peak [101] of the TiO_2_ phase.

### Cyclic voltammetry

**Fig. 3:**
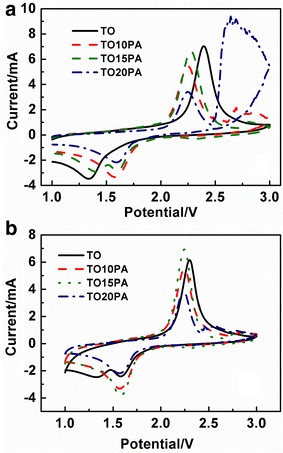
Cyclic voltammograms on TiO_2_ and TiO_2_/PANI composites at a scan rate of 1 mVs^−1^ for the first (**a**) and third cycles (**b**)

Figure 3 shows the comparison of cyclic voltammograms of TO/PA composite materials with TO at a scan rate of 1mVs^−1^ for the first (Fig. 3a) and second cycles (Fig. 3b). A reversal system showing both the anodic and cathodic peaks are presented in Fig. 3, with the peaks respectively at 2.4 V/1.3 V for TO and 2.3 V/1.6 V for TO/PA. In a typical TiO_2_/Li half-cell, the electrochemical process is as follows: TiO_2_ + x Li^+^ + xe^−^ LixTiO_2_ (Wang et al. 2007).

The maximum number of inserted Li^+^ was evaluated to be 0.5 (Nuspl et al. 1997) that result in a theoretical capacity of 167.5 mAh g^−1^ (Lou and Archer 2008). The cathodic peaks at 1.6 V (TO15PA) and 1.3 V (TO) determines that two phase transitions happened on the structure from tetragonal anatase to orthothombic Li_0.5_TiO_2_ when the insertion coefficient X in reaction (Wang et al. 2007) has reached about 0.5 (Chen and Lou 2010; Sivakkumar and Kim 2007). There is a coupling of cathodic/anodic peaks around 2.8 V, especially on TO20PA which is associated with the doping/leaching processes of polyaniline (Kavan et al. 1996).

The results of CV are listed in Table 1. The onset potentials of TO/PA composites (see Fig. 3a) compared with those of TO were more negative compared with that exhibited by TO/PA composites on the first and second cycles, which have demonstrated that TO/PA composites have a higher electrochemical activity than pure TO. It can also be observed that the current of peak increases slightly on TO/PA on second cycles (see Table 1), indicating a possible activates process in the electrode material, and suggesting the high reversibility of the insertion/extraction reactions. However, on TO20PA, the peak at 2.8 V (PANI) has dramatically decreased on the second scans which indicate that PANI is less stable.

**Table 1:** Summary of CV results (Fig. 3) on TO and TO/PANI composites at a scan rate of 1 mVs^−1^ for the first and second cycles

Cycle number	TO		TO10PA		TO15PA		TO20PA	
	Potential (V)	Current (mA)	Potential (V)	Current (mA)	Potential (V)	Current (mA)	Potential (V)	Current (mA)
1th	2.39	7.0	2.25	5.5	2.27	6.6	2.24	3.4
2th	2.29	6.1	2.25	5.6	2.25	6.9	2.23	3.8

### Battery performance

**Fig. 4:**
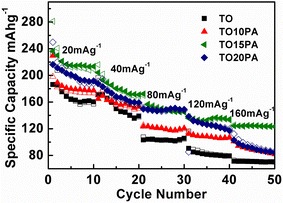
Specific capacity vs. cycle number for TiO_2_ and TiO_2_/PANI composites at different current rates

The capacity vs. cycle number profiles of TO/PANI composites at different current density are shown in Fig. 4. Pure PANI tested in conditions similar to TO/PANI has shown negligible capacity. The capacities were calculated based on the mass of TO composites. As shown in Fig. 4, on first cycle the TO15PA displayed the highest capacity of 281 mAh g^−1^, while the TO10PA, TO20PA and TO showed relatively lower capacity of 198, 250, and 230 mAh g^−1^ respectively at the current rate of 20 mAg^−1^. The TO15PA still keeps the highest capacity of 210 mAh g^−1^ among the materials at the end of the tenth cycle; the TO10PA and TO20PA displayed capacity of 177 and 188 mAh g^−1^. The lowest capacity of 157 mAh g^−1^ is shown on TO.

During the rate performance, the TO/PANI composites exhibited better capacity retention when the current rate eventually increased to 160 mAg^−1^. The capacities of pure TO reduced to 70.1 mAh g^−1^ at a current rate of 160 mAg^−1^ (at the end of tenth cycle), which is 30 % retention of this at the initial stage. The TO10PA, TO15PA and TO20PA showed the capacities of 83.1, 123.1 and 85.9 mAh g^−1^ respectively which are 42, 44 and 34 % of the capacities of the initial stage at a current rate of 20 mAg^−1^. This indicates the improved capacity rate of TO/PANI composites compared with pure TO.

**Fig. 5:**
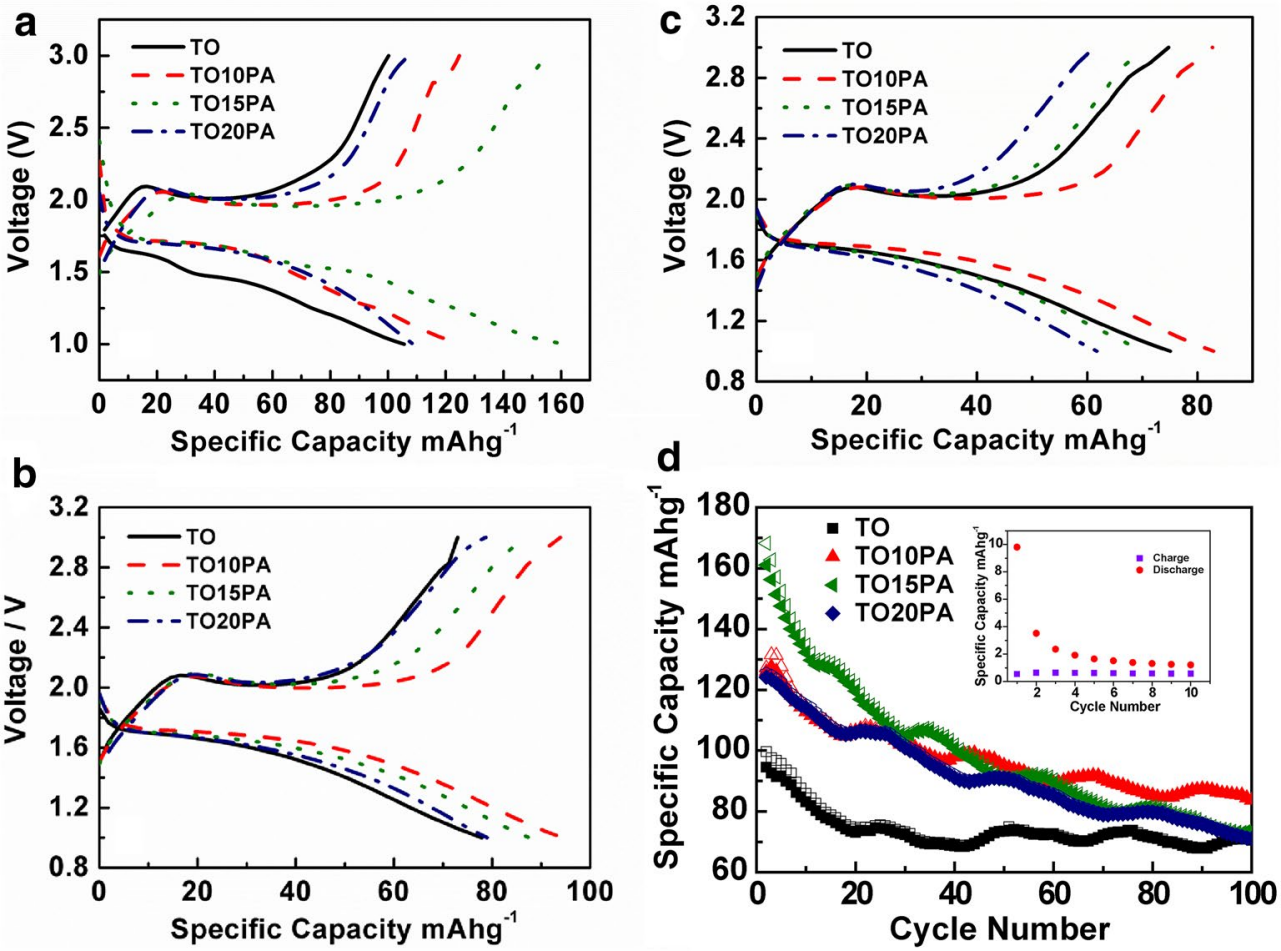
Cell voltage as a function of specific capacity on TiO_2_, PANI (Insert of d) and TiO_2_/PANI composites as anode at a current rate of 200 mAg^−1^ (**a** 2th cycle; **b** 5th cycle; **c** 10th cycle; **d** specific capacity vs cycle number)

For TO/PANI composites, the PANI might keep the TO particles electrically connected and offers conductive pathways between the active particles, the substrate and the electrolyte. Thus it facilitates the fast charge transfer. Figure 5 presented battery properties of TO/PANI composites at the current rate of 200 mAg^−1^ within a voltage range of 1.0–3.0 V during 100 consecutive cycles. These are consistent with typical charge/discharge voltage profiles of TO. Specific capacity and capacity retention at a current rate of 200 mAg^−1^ were summarized in Table 2. As presented in Fig. 5a, the TO15PA has achieved the highest lithiation capacity of 168.2 mAhg^−1^ and de-lithiation capacity of 161.0 mAhg^−1^ in the second cycle. TO, TO10PA and TO20PA showed the capacities of 99.6, 127.2 and 125.4 mAh g^−1^ respectively. The order of capacity decrease was TO15PA > TO10PA ≈ TO20PA > TO. However, the capacities of TO, TO10PA, TOPA and TO20PA were reduced respectively to 73.5, 95.7, 91.2 and 91.2 mAh g^−1^ at the end of 50 cycles (Fig. 5b). The capacities further decreased respectively to 70.8, 83.7, 73.5 and 71.3 mAh g^−1^ at the end of 100 cycles (Fig. 5c). The reversible capacities fade of TO, TO10PA, TOPA and TO20PA from the second cycle to the 100th cycle were about 0.3, 0.4, 0.9 and 0.5 per cycle and retention of capacities on TO, TO10PA, TO15PA and TO20PA were 71.1, 65.8, 43.7 and 56.8 % after 100 cycles respectively (see Fig. 5d and Table 2). It is observed from these results that TO10PA exhibited the highest capacity and cycle stability after 100 cycles though TO15PA had the highest initial capacity. TO15PA and TO20PA have shown poor cycle stability after 50 cycles. That can be related to the degradation of polyaniline and poor electrochemical stability in the presence of liquid electrolyte (Novák et al. 1997; Kavan et al. 1996). The charge/discharge Performance of PANI was displayed inset of Fig. 5d, the PANI had very low capacity at the current rate of 200 mAg^−1^, which showed PANI probably have played the role of increased conductivity of the composites.

**Table 2:** Summary of specific capacity and capacity retention at a current rate of 200 mAg^−1^ with different cycle number (1th, 50th and 100th)

Cycle number	TO		TO10PA		TO15PA		TO20PA	
	Capacity (mAg^−1^)	Capacity retention (%)	Discharge (mAg^−1^)	Capacity retention (%)	Discharge retention (mAg^−1^)	Capacity retention (%)	Discharge (mAg^−1^)	Capacity retention (%)
1st	99.6	–	127.2	–	168.2	–	125.4	–
50th	73.5	73.7	95.2	74.8	91.2	54.2	91.2	72.7
100th	70.8	71.1	83.7	65.8	73.5	43.7	71.3	56.8

## Conclusion

In this work, TO/polyaniline composites were prepared via the solid coating method. The results demonstrated that polyaniline has effectively enhanced the reversible capacity of commercial TO. At the current rate of 20 mAg^−1,^ the TO15PA has showed the highest capacity of 281 mAh g^−1^, while the capacities TO10PA, TO20PA and TO were 198, 250, and 230 mAh g^−1^ respectively. In addition, TO, TO10PA, TO15PA and TO20PA showed the capacities of 99.6, 127.2, 168.2 and 125.4 mAh g^−1^ respectively at the current rate of 200 mAg^−1^. TO10PA retained the best capacity (83.7 mAh g^−1)^ and cycle stability after 100 cycles. It is suggested that polyaniline is a potential matrix material for lithium ion batteries; however, the synthesis of polyaniline or polyaniline composites still need improvement to meet the requirements for lithium ion battery applications.

## References

[CR1] Chen JS, Lou XW (2010). The superior lithium storage capabilities of ultra-fine rutile TiO _2_ nanoparticles. J Power Sources.

[CR2] Chen M, Du C, Wang L (2012). Silicon/Graphite/Polyaniline Nanocomposite with Improved Lithium-Storage Capacity and Cyclability as Anode Materials for Lithium-ion Batteries. Int J Electrochem Soc.

[CR3] Chew S, Guo Z, Wang J (2007). Novel nano-silicon/polypyrrole composites for lithium storage. Electrochem Commun.

[CR4] Dong Z, Zhang J, Zhao X (2013). Sulfur@ hollow polypyrrole sphere nanocomposites for rechargeable Li–S batteries. RSC Advances.

[CR5] Fattakhova Rohlfing D, Wark M, Brezesinski T (2007). Highly organized mesoporous TiO2 films with controlled crystallinity: a Li-insertion study. Adv Funct Mater.

[CR6] Huang Y, Goodenough JB (2008). High-rate LiFePO_4_ lithium rechargeable battery promoted by electrochemically active polymers. Chem Mater.

[CR7] Jeong J, Choi BG, Lee SC (2013). Hierarchical hollow spheres of Fe_2_O_3_@ polyaniline for lithium ion battery anodes. Adv Mater.

[CR8] Jiang C, Wei M, Qi Z (2007). Particle size dependence of the lithium storage capability and high rate performance of nanocrystalline anatase TiO_2_ electrode. J Power Sources.

[CR9] Karthikeyan K, Amaresh S, Aravindan V (2013). Li (Mn _1/3_ Ni _1/3_ Fe _1/3_) O _2_–Polyaniline hybrids as cathode active material with ultra-fast charge–discharge capability for lithium batteries. J Power Sources.

[CR10] Kavan L, Kratochvilová K, Grätzel M (1995). Study of nanocrystalline TiO_2_ (anatase) electrode in the accumulation regime. J Electroanal Chem.

[CR11] Kavan L, Grätzel M, Rathouský J (1996). Nanocrystalline TiO2 (anatase) electrodes: surface morphology, adsorption, and electrochemical properties. J Electrochem Soc.

[CR12] Kavan L, Rathouský J, Grätzel M (2000). Surfactant-templated TiO_2_ (anatase): characteristic features of lithium insertion electrochemistry in organized nanostructures. J Phys Chem B.

[CR13] Lai C, Li G, Dou Y (2010). Mesoporous polyaniline or polypyrrole/anatase TiO_2_ nanocomposite as anode materials for lithium-ion batteries. Electrochim Acta.

[CR14] Lai C, Zhang H, Li G (2011). Mesoporous polyaniline/TiO_2_ microspheres with core–shell structure as anode materials for lithium ion battery. J Power Sources.

[CR15] Liang R, Cao H, Qian D (2011). Designed synthesis of SnO_2_-polyaniline-reduced graphene oxide nanocomposites as an anode material for lithium-ion batteries. J Mater Chem.

[CR16] Lou XW, Archer LA (2008). A general route to nonspherical anatase TiO_2_ hollow colloids and magnetic multifunctional particles. Adv Mater.

[CR17] Novák P, Müller K, Santhanam K (1997). Electrochemically active polymers for rechargeable batteries. Chem Rev.

[CR18] Nuspl G, Yoshizawa K, Yamabe T (1997). Lithium intercalation in TiO_2_ modifications. J Mater Chem.

[CR19] Sivakkumar S, Kim D (2007). Polyaniline/carbon nanotube composite cathode for rechargeable lithium polymer batteries assembled with gel polymer electrolyte. J Electrochem Soc.

[CR20] Su X, Wu Q, Zhan X (2012). Advanced titania nanostructures and composites for lithium ion battery. J Mater Sci.

[CR21] Sudant G, Baudrin E, Larcher D (2005). Electrochemical lithium reactivity with nanotextured anatase-type TiO 2. J Mater Chem.

[CR22] Tang Y, Yang L, Qiu Z (2009). Template-free synthesis of mesoporous spinel lithium titanate microspheres and their application in high-rate lithium ion batteries. J Mater Chem.

[CR23] Wang J, Polleux J, Lim J (2007). Pseudocapacitive contributions to electrochemical energy storage in TiO_2_ (anatase) nanoparticles. J Phys Chem C.

[CR24] Wang W, Sa Q, Chen J (2013). Porous TiO2/C nanocomposite shells as a high-performance anode material for lithium-ion batteries. ACS Appl Mater Interfaces.

[CR25] Yang HG, Zeng HC (2004). Preparation of hollow anatase TiO_2_ nanospheres via Ostwald ripening. J Phys Chem B.

[CR26] Yang HG, Sun CH, Qiao SZ (2008). Anatase TiO_2_ single crystals with a large percentage of reactive facets. Nature.

[CR27] Yang Z, Du G, Meng Q (2012). Synthesis of uniform TiO _2_@ carbon composite nanofibers as anode for lithium ion batteries with enhanced electrochemical performance. J Mater Chem.

[CR28] Zeng X, Ko T (1998). Structures and properties of chemically reduced polyanilines. Polymer.

